# Melioidosis: an emerging infection in Taiwan?

**DOI:** 10.3201/eid0703.010310

**Published:** 2001

**Authors:** P R Hsueh, L J Teng, L N Lee, C J Yu, P C Yang, S W Ho, K T Luh

**Affiliations:** National Taiwan University Hospital, Taipei, Taiwan.

## Abstract

From January 1982 to May 2000, 17 infections caused by Burkholderia pseudomallei were diagnosed in 15 patients in Taiwan; almost all the infections were diagnosed from 1994 to May 2000. Of the 15 patients, 9 (60%) had underlying diseases, and 10 (67%) had bacteremic pneumonia. Thirteen (76%) episodes of infection were considered indigenous. Four patients died of melioidosis. Seventeen B. pseudomallei isolates, recovered from eight patients from November 1996 to May 2000, were analyzed to determine their in vitro susceptibilities to 14 antimicrobial agents, cellular fatty acid and biochemical reaction profiles, and randomly amplified polymorphic DNA patterns. Eight strains (highly related isolates) were identified. All isolates were arabinose non-assimilators and were susceptible to amoxicillin-clavulanate, piperacillin-tazobactam, imipenem, and meropenem. No spread of the strain was documented.


### Melioidosis: An Emerging Infection in Taiwan?

**Po-Ren Hsueh,* Lee-Jene Teng,* Li-Na Lee,*† Chong-Jen Yu,* Pan-Chyr Yang,* Shen-Wu Ho,*† and Kwen-Tay Luh,***
*National Taiwan University Hospital, Taipei, Taiwan; and
†National Taiwan University College of Medicine, Taipei, Taiwan

From January 1982 to May 2000, 17 infections caused by *Burkholderia pseudomallei* were diagnosed in 15 patients in Taiwan; almost all the infections were diagnosed from 1994 to May 2000. Of the 15 patients, 9 (60%) had underlying diseases, and 10 (67%) had bacteremic pneumonia. Thirteen (76%) episodes of infection were considered indigenous. Four patients died of melioidosis. Seventeen *B. pseudomallei* isolates, recovered from eight patients from November 1996 to May 2000, were analyzed to determine their in vitro susceptibilities to 14 antimicrobial agents, cellular fatty acid and biochemical reaction profiles, and randomly amplified polymorphic DNA patterns. Eight strains (highly related isolates) were identified. All isolates were arabinose non-assimilators and were susceptible to amoxicillin-clavulanate, piperacillin-tazobactam, imipenem, and meropenem. No spread of the strain was documented.

Melioidosis is an infectious disease of humans and animals caused by *Burkholderia pseudomallei* (1). This organism is widely distributed in rice field soil and in stagnant water throughout the tropics (1-8). Although a major disease in Southeast Asia and northern Australia, melioidosis occurs sporadically throughout the world (often in patients with a history of residence in these disease-endemic areas) (1). Humans are usually infected by traumatic inoculation of the organism from the soil or, rarely, by inhalation or ingestion (1,7). Clinical manifestations are protean, ranging from benign and localized abscess, to severe, community-acquired pneumonia to fatal septicemia (1,3,4,7). The two biotypes of *B. pseudomallei* are categorized by their ability to assimilate L-arabinose (9). The arabinose non-assimilators (Ara-) are virulent and can be isolated from both clinical specimens and the environment, whereas the arabinose assimilators (Ara+) are usually avirulent and mainly found in the environment (9,10). Relapse, recrudescence, or reinfection may occur in immunocompromised patients under inappropriate antimicrobial treatment or after resolution of primary infection (1,7,11,12).

In Taiwan in 1985, melioidosis was first reported in a previously healthy patient with multilobar pneumonia, which developed secondary to his near drowning in the Philippines (13). Since then, only six other cases have been reported (14-18). In our study, an additional eight patients with melioidosis found from 1996 to 2000 by National Taiwan University Hospital (NTUH) were reported and microbiologic characteristics (including biotyping, cellular fatty acid chromatograms, antimicrobial susceptibilities, and genotyping) of the strains isolated from these patients were evaluated.

Address for correspondence: Kwen-Tay Luh, Department of Laboratory Medicine, National Taiwan University Hospital, No. 7 Chung-Shan South Road, Taipei, Taiwan; fax: 886-2-23224263; e-mail: luhkt@ha.mc.ntu.edu.tw

### Materials and Methods

### Patients and Bacterial Isolates

From January 1980 to May 2000, eight patients with melioidosis were treated at NTUH, a tertiary-care referral center with 2,000 beds in northern Taiwan, and seven patients reported to have melioidosis were treated at other hospitals in Taiwan. Data on demographics, underlying diseases, travel history, type of infection, antimicrobial treatment, and outcome of these patients were analyzed. Infections were considered indigenous if patients had no history of residence or travel to China, Australia, or Southeast Asia. A total of 17 isolates of *B. pseudomallei* from various clinical specimens of the eight patients treated at NTUH were studied. These isolates were identified by conventional biochemical methods and confirmed by the API 20NE identification system (Biomerieux, Basingstoke, UK) (19,20).

### Susceptibility and Antibiotypes

The following antimicrobial agents were provided by their manufacturers for use in this study: amoxicillin-clavulanate (SmithKline Beecham, Welwyn Garden City, UK); cefotaxime (Marion Merrell Dow, Cincinnati, OH); ceftazidime (Glaxo, Greenford, UK); amoxicillin, cefepime, aztreonam, and amikacin (Bristol-Meyer Squibb, Princeton, NJ); imipenem (Merck Sharp & Dohme, Rahway, NJ); meropenem (Sumitomo Pharmaceuticals, Osaka, Japan); piperacillin-tazobactam (Wyeth-Ayerst Laboratories, Pearl River, NY); flomoxef (Shionogi, Osaka, Japan); trovafloxacin (Pfizer Inc., New York, NY); and ciprofloxacin and moxifloxacin (Farbenfabriken Bayer GmbH, Leverkrusen, Germany). MICs of these antimicrobial agents were determined by the agar dilution method according to guidelines established by the National Committee for Clinical Laboratory Standards (21). Isolates were grown overnight on trypticase soy agar plates supplemented with 5% sheep blood (BBL Microbiology Systems, Cockeysville, MD) at 37°C.

Bacterial inocula were prepared by suspending the freshly grown bacteria in sterile normal saline and adjusted to a 0.5 McFarland standard. Mueller-Hinton agar (BBL Microbiology Systems) was used for susceptibility testing. With a Steers replicator, an organism density of $10^4$ CFU/spot was inoculated onto the appropriate plate with various concentrations of antimicrobial agents. *Escherichia coli* ATCC 25922 and *Pseudomonas aeruginosa* ATCC 27853 were included as control strains. Antibiotypes were considered identical if all MICs tested were identical or within a twofold dilution discrepancy (22).

### Cellular Fatty Acid Chromatogram and Biotypes

Biotypes of these isolates were identified according to the reaction profiles obtained by API 20NE. Arabinose utilization was determined by growth on minimal salt agar containing L-arabinose (0.2%) (9). Fatty acid methyl esters (FAMEs) of these isolates were analyzed by gas-liquid chromatography using a Hewlett-Packard 5890A (Hewlett-Packard; Avondale, PA) as described previously (22). The software library used to identify the *B. pseudomallei* was TSA, version 3.9 (Microbial ID Inc., Newark, DE).

### Strain Typing

Extraction of genomic DNA and polymerase chain reaction (PCR) for determining random amplified polymorphic DNA (RAPD) patterns generated by arbitrarily primed PCR of the 17 isolates of *B. pseudomallei* were performed as previously described (22). Three oligonucleotide primers used were: M13 (5'-TTATGTAAAACGACGGCCAG-3' (Gibco BRL products, Gaithersburg, MD), ERIC1 (5'-GTGAATCCCCAGGAGCTTACAT-3' (Gibco Bethesda Research Laboratories Products), and OPH-03 (5'-AGACGTCCAC-3') (Operon Technologies, Inc., Alameda, CA). To interpret RAPD patterns, both faint and intense bands were included (22). The entire procedure, from bacterial growth to interpretation of RAPD pattern, was repeated three times for each isolate to confirm results. Patterns were considered identical only if they differed by no more than one band. Isolates were defined as being of the same strain (highly related isolates) if they had identical antibiotypes, biotypes, and RAPD patterns.

### Results

### Clinical Characteristics of Patients

From January 1982 to May 2000, 17 episodes of infection caused by *B. pseudomallei* were diagnosed in 15 patients in Taiwan. All but one episode occurred between 1994 and 2000 (Figure 1; Table 1), indicating that cases have increased substantially in recent years. In these 15 patients, 13 were male; mean age was 64 years (range, 40 to 76 years). Patient 1 acquired pneumonia secondary to his near drowning in the Philippines. Patient 4 had had a fever during his stay in mainland China. Patient 6 had fever and left upper abdominal pain on his arrival in Taiwan after a 4-day trip in Rangoon, Burma. Patient 8 had septicemic melioidosis 5 years after travel to Thailand. The 13 other episodes (76%) were considered indigenous. Of the 11 patients with indigenous melioidosis, occupation was known for 7 patients (patients 9 to 15); none were rice farmers. Of the 15 infected patients, 9 (60%) had underlying diseases (6 had diabetes mellitus, and 3 had chronic obstructive pulmonary disease),

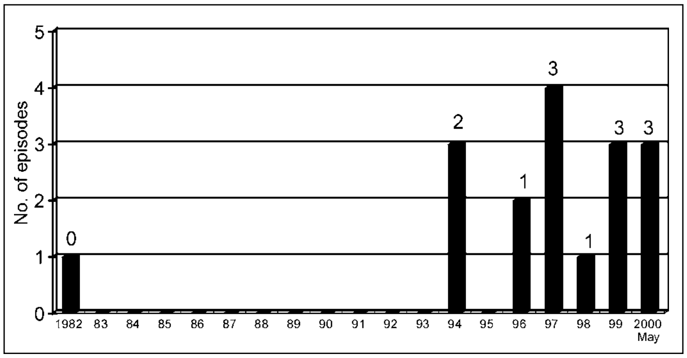

Figure 1. Cases of melioidosis in Taiwan, by year of diagnosis. Number above each bar indicates number of episodes with indigenous infection.

12 patients (80%) had pneumonia (including 10 with bacteremia and 1 with concomitant peritonitis), 2 (13%) had soft-tissue abscess, and 1 (7%) had mycotic aneurysm. Two patients each had two episodes of infection, separated by 8 and 10 months, respectively. Four patients (27%) died of melioidosis. One patient (no. 15), who had pneumonia caused by an organism initially identified as *P. aeruginosa* and treated at another hospital with ceftazidime and amikacin for 1 month, died on the third day in our hospital of refractory pneumonia complicated by empyema and acute respiratory distress syndrome. Two sets of blood cultures collected upon admission grew *B. pseudomallei*. Susceptibility results by the routine disk susceptibility test showed the organism was resistant to ceftazidime and amikacin. MICs of the organism to ceftazidime and amikacin were 32 µg/mL and >256 µg/mL, respectively (Table 2).

### Bacterial Isolates and Biotypes

All *B. pseudomallei* isolates had characteristic colonial morphology on trypticase soy agar supplemented with 5% sheep blood (BBL Becton Dickinson, Microbiology, Cockeysville, MD), positive oxidase reaction, and resistance to colistin and gentamicin. Of the 17 isolates (from eight patients), two biochemical profiles based on the results of identification by the API 20NE system were 1156576 (biotype I; citrate negative) and 1156577 (biotype II; citrate positive). All isolates tested were Ara-. The probability of identification of each *B. pseudomallei* biochemical profiles was 99.9%.

All 17 isolates (from eight patients) studied had similar FAME profiles, and all had five major FAMEs (each presenting >3% of the total): 14:0 (tetradecanoic acid), 3-OH-14:0 (3-hydroxytetradecanoic acid), 16:0 (hexadecanoic acid), 17:0cyc (-cis-9, 10-methylenehexadecanoic acid), 2-OH-16:0 (2-hydroxyhexadecanoic acid), 3-OH-16:0 (3-hydroxyhexadecanoic acid), 18:1w7c (cis-11-octadecenoic acid), and 19:0cyc (-cis-11, 12-methyleneoctadecanoic acid). FAME profiles of these isolates were consistent with the identification of *B. pseudomallei.*

### Antimicrobial Susceptibilities and Antibiotypes

MICs of 14 antimicrobial agents for the 17 isolates of *B. pseudomallei* were determined (Table 2). When MIC breakpoints for susceptibility and resistance used for non-*Enterobacteriaceae* were applied to *B. pseudomallei* (21), all isolates were susceptible to amoxicillin-clavulanate,

Table 1. Clinical characteristics of 15 patients in Taiwan with invasive infections caused by *Burkholderia pseudomallei*

| Patient no./ref | Age/ sex | Travel history | Underlying diseases/ associated conditions | Clinical diagnosis | Isolate source (date) | Treatment | Outcome |
|---|---|---|---|---|---|---|---|
| 1 (13) | 46/M | Philippines | Near drowning | Cavitary pneumonia | Blood (9/82) | Cephalothin, amikacin (30 d) | Surv |
| 2 (14) | 75/F | None | Liver cirrhosis, uremia, DM | Pneumonia, peritonitis | Blood, ascites (8/94) | Cefazolin, gentamicin (1 d) | Died |
| 3 (14) | 70/M | None | None | Pneumonia | Blood (8/94) | Ceftazidime (30 d), amoxicillin-clavulanate (3 mo) | Surv |
| 4 (15) | 70/M | China | None | Mycotic aneurysm | Blood (11/94); aortic tissue (12/94) | Ceftazidime (35 d); amoxicillin-clavulanate (6 mo) | Surv |
| 5 (16) | 75/M | None | DM | Pneumonia | Blood (11/96) | Ceftazidime (14 d); amoxicillin-clavulanate (2 mo) | Surv |
| 6 (17) | 51/M | Burma | DM | Pneumonia, adrenal gland abscess | Blood (7/97) | Ceftazidime (2 d); cotrimoxazole (60 d) | Surv |
| 7 (18) | 40/M | None | DM | Pneumonia, ARDS | Blood (7/97) | Moxalactam (9 d); netilmicin (9 d); erythromycin (9 d) | Died |
| 8 (PR) | 56/M | Thailand | None | Pneumonia | Blood-A (11/96) | Ceftazidime, gentamicin (1 d) | Died |
| 9 (PR) | 67/M | None | COPD | Pneumonia | Blood-B (1/97) | Ceftazidime (14 d); amoxicillin-clavulanate (2 d) | Surv |
| 10 (PR) | 73/M | None | Prostate cancer with bone metastasis; DM; cyproterone and leuprolide acetate use | Cavitary pneumonia | Lung aspirate-C1 7/97); RAPDa patterns sputum-C2 (7/97) | Ciprofloxacin (5 mo) | Surv |
| 11 (PR) | 76/F | None | None | Septic arthritis, subcutaneous abscess | Blood-D1; blood-D2; synovial fluid-D3; abscess fluid-D4; wound drainage-D5; all 9/98 | Ciprofloxacin (4 mo); surgical drainage | Surv |
| 12 (PR) | 58/M | None | None | Subcutaneous abscess | Abscess fluid-E1 (4/99); abscess fluid-E2 (2/2000) | Piperacillin (10 d); ciprofloxacin (14 d) | Surv |
| 13 (PR) | 66/M | None | DM | Pneumonia, arthritis, subcutaneous abscess | Lung aspirate-F1 (5/99); synovial fluid-F2 (5/99); abscess aspirate-F3 (1/2000) | Imipenem (10 d); ciprofloxacin (2 mo); meropenem (1 mo); amoxicillin-clavulanate (4 mo) | Surv |
| 14 (PR) | 74/M | None | COPD | Pneumonia | Blood-G (9/99) | Ceftazidime, amikacin (14 mo); ciprofloxacin (2 mo) | Surv |
| 15 (PR) | 70/M | None | COPD, TB | Pneumonia, empyema; ARDS | Blood-H1 Blood-H2 (1/2000) | Ceftazidime, amikacin (1 mo) | Died |

Abbreviations used in this table: ARDS, adult respiratory distress syndrome; COPD, chronic obstructive pulmonary disease; d, day; DM, diabetes mellitus; TB, tuberculosis; mo, month; PR, present report; surv, survived.

piperacillin-tazobactam, imipenem, and meropenem. Most isolates were intermediate or resistant to ampicillin, flomoxef, cefepime, aztreonam, amikacin, and ciprofloxacin. Ceftazidime had in vitro activity equal to or greater than that of cefotaxime against *B. pseudomallei*. Two isolates (both from patient 12) showed high resistance to ceftazidime and cefepime and intermediate resistance to cefotaxime. Five antibiotypes (antibiotypes I to V) were identified in the 17 isolates.

### RAPD Patterns and Identification

Eight RAPD patterns were identified by use of the three primers (Figure 2). RAPD patterns of multiple isolates from the same patients were identical. The two isolates (recovered 10 months apart) from patient 10 belonged to strain 5; two of the three isolates (recovered 8 months apart) from patient 11 were also identical (strain 6; Table 3). Isolates recovered from different patients had distinct RAPD patterns.

Table 2. Susceptibilities and antibiotypes of 17 isolates of *Burkholderia pseudomallei* isolated in Taiwan from November 1996 to January 2000

| Patient no./ isolate | MIC (mg/mL)[a] AM | AMC | PZP | CTX | CAZ | FEP | FLO | ATM | IPM | MEM | AN | CIP | TRO | MOX | Antibiotic type |
|---|---|---|---|---|---|---|---|---|---|---|---|---|---|---|---|
| 8/A | 64 | 8 | 0.5 | 4 | 4 | 16 | 128 | 16 | 0.5 | 1 | 64 | 2 | 2 | 2 | I |
| 9/B | 64 | 8 | 1 | 4 | 4 | 16 | 128 | 16 | 0.5 | 1 | 128 | 2 | 2 | 2 | I |
| 10/C1, 2 | 64 | 8 | 0.5 | 4 | 4 | 16 | 128 | 16 | 0.5 | 1 | 32 | 1 | 2 | 2 | II |
| 11/D1, 2, 3, 4, 5 | 64 | 8 | 0.5 | 2 | 1 | 16 | 128 | 16 | 0.5 | 1 | 32 | 2 | 4 | 1 | III |
| 12/E1, 2 | 64 | 8 | 0.5 | 2 | 1 | 8 | 128 | 16 | 0.5 | 1 | 32 | 2 | 4 | 1 | III |
| 13/F1, 2, 3 | 64 | 8 | 0.5 | 2 | 1 | 16 | 128 | 16 | 0.5 | 1 | 16 | 2 | 4 | 1 | III |
| 14/G | 16 | 2 | 0.12 | 2 | 1 | 8 | 64 | 8 | 0.25 | 0.5 | 64 | 1 | 1 | 1 | IV |
| 15/H1, 2 | 64 | 8 | 1 | 16 | 32 | 64 | 128 | 32 | 0.5 | 2 | >256 | 4 | 8 | 4 | V |

[a]AM, amoxicillin; AMC, amoxicillin-clavulanate; PZP, piperacillin-tazobactam; CTX, cefotaxime; CAZ, ceftazidime; FEP, cefepime; FLO, flomoxef; ATM, aztreonam; IPM, imipenem; MEM, meropenem; AN, amikacin; CIP, ciprofloxacin; TRO, trovafloxacin; MOX, moxifloxacin.

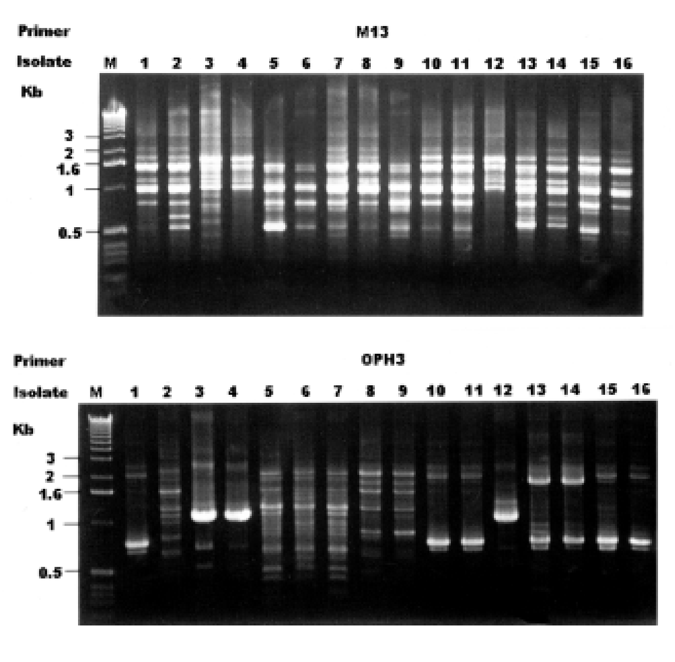

Figure 2. Random amplified polymorphic DNA (RAPD) patterns of 16 isolates of *Burkholderia pseudomallei* generated by arbitrarily primed polymerase chain reaction with the primers M13 (upper panel) and ERIC1 (lower panel). Lanes: M, molecular size marker; 1, isolate A; 2, isolate B; 3 and 4, isolates C1 and C2; respectively; 5 to 9, isolates D1 to D5; 10 and 11, isolates E1 and E2, respectively; 12, isolates G; 13 and 14, isolates F1 and F2, respectively; and 15 and 16, isolates H1 and H2, respectively. (See Table 3 for designation of isolates).

Table 3. Phenotypic and genotypic characteristics of 17 isolates of *Burkholderia pseudomallei*

| Isolate(s)/ patient no. | Anti-biotype | Biotype | RAPD[a] patterns M13/ERIC1/ OPH-3 | Strain |
|---|---|---|---|---|
| A/8 | I | A | a | 1 |
| B/9 | I | A | b | 2 |
| C1, C2/10 | II | A | c | 3 |
| D1, D2, D3, D4, D5/11 | III | A | d | 4 |
| E1, E2/12 | III | A | e | 5 |
| F1, F2, F3/13 | III | A | f | 6 |
| G/14 | IV | B | g | 7 |
| H1, H2/15 | V | A | h | 8 |

[a]RAPD = Random amplified polymorphic DNA.

### Conclusion

Between January 1982 and 1994, one episode of melioidosis was identified in Taiwan. From 1994 to May 2000, 16 more cases occurred. Whether these figures represent an actual increase in *B. pseudomallei* infections in Taiwan or better recognition of this organism by microbiology laboratories is difficult to clarify. In NTUH, the first clinical isolate of *B. pseudomallei* was recognized in 1980 (the medical record and isolate are now unavailable). Since then, improved laboratory procedures and increasing alertness of laboratory staffs permit more accurate identification of this organism. However, no *B. pseudomallei* isolate was identified in our laboratory from 1981 to 1995. Thus, from our vantage point, the observed increase in cases of melioidosis is indeed an emerging problem of the last 5 years.

Most of these infections were indigenous. All strains, whether imported or indigenous, were genetically distinct. Different patients were infected with different strains, indicating that spread of strains (intercontinental or within this island), as with *Penicillium marneffei* (another emerging pathogen in Taiwan), did not occur (23). Our data suggest that Taiwan should be considered a melioidosis-endemic area, in addition to China, Australia, and Southeast Asia.

Melioidosis has been called the great mimicker because of its protean clinical features (1,7). The most common clinical sign is an acute pulmonary infection (as in our study), though its chronic pulmonary form often resembles tuberculosis (3,16). When localized, melioidosis may cause abscess formation in skin, soft tissue, joints, and visceral organs (1). Melioidosis can become a latent infection that later (as much as 26 years after initial exposure) reactivates into a full-blown illness (even with acute septicemia) (1,12). The content of Ara- *B. pseudomallei* in the soil of a geographic area has been documented to correlate directly with the incidence of melioidosis in that area (5,6). In our study, all but four patients had no prior exposure to well-known disease-endemic areas. Therefore, the strains of *B. pseudomallei* they acquired might have originated in Taiwanese soil. Unlike other reports (24), most (87%) of our patients were male. Also, all but one patient with indigenous infection were >65 years, and none were rice farmers or obviously had heavy exposure to soil. Environmental surveys for the presence of this organism in the soil of Taiwan, especially in rice fields, are ongoing; thus far, *B. pseudomallei* has been isolated from two soil samples (data not shown). Although Ara+ *B. pseudomallei* has been reported to cause severe infection (10), all isolates causing melioidosis in our study were Ara-. Our findings support previous observations (1,5,6).

*B. pseudomallei* are frequently intrinsically resistant to many antibiotics, including aminoglycosides and first- or second-generation cephalosporins (25,26). Current recommendations for therapy of severe melioidosis include intravenous ceftazidime or imipenem for 10 days to 4 weeks (25,26), followed by maintenance therapy with oral amoxicillin-clavulanate or a combination of trimethoprim-sulfamethoxazole and doxycycline for 10 to 18 weeks (27-31). Cefotaxime and ceftriaxone are both less active than ceftazidime against *B. pseudomallei* in vivo and in vitro (28). However, the observation of ceftazidime resistance's emerging during treatment has been previously reported (30). Its occurrence in our patient 15 might be related to the presence of an empyema. In areas in which melioidosis is endemic, empirical regimens that contain cefotaxime or ceftriaxone for the treatment of severe community-acquired pneumonia or septicemia may not be appropriate.

Some investigators suggest that melioidosis is a facultative intracellular infection (25). The failure of beta-lactam antibiotics to penetrate intracellular sites and kill nonmultiplying dormant bacteria may explain the frequent relapses of melioidosis after treatment with these drugs (25). On the other hand, relapse is documented to be less common (10% versus 30%) in patients who complete a full course of antibiotic treatment (32). Our two patients who had recurrent infections both received oral ciprofloxacin for 2 or 8 weeks. Nearly all isolates had MICs ranging from 1 µg/mL to 4 µg/mL for the three newer fluoroquinolones tested. Further studies are needed to determine the clinical efficacy of these newer fluoroquinolones for treating melioidosis and their role in preventing future relapse.

Several molecular typing methods have been applied to *B. pseudomallei* to evaluate the strain relationship in isolates recovered from humans and environment (33-37). Among these methods, RAPD typing has also been documented to be useful for analyzing isolates that cause recurrent infection or reinfection (35). In our study, RAPD typing using three primers clearly demonstrated the genetic diversity of isolates from different patients (with either imported or indigenous infections). In addition, this method showed that multiple isolates from the same patient and isolates causing recurrent infections were genetically identical. The microbial identification system, based on cellular FAME analysis by use of gas chromatography, is an established method for identifying species of bacteria and fungi and showing clustering in bacterial and fungal strains (22,23). Although all our isolates of *B. pseudomallei* had identical FAME profiles, cluster analysis of these esters (data not shown) failed to provide acceptable discriminatory power for typing the isolates.

In conclusion, Taiwan should be included as an endemic area of melioidosis, and physicians managing patients in Taiwan should be alert to the possibility that this organism might cause community-acquired pneumonia and sepsis.

Dr. Hsueh is assistant professor, departments of Laboratory Medicine and Internal Medicine, National Taiwan University College of Medicine, Taipei, Taiwan. His research interests include epidemiology of emerging and nosocomial infections and mechanisms of antimicrobial drug resistance. He is actively involved in developing a national research program for antimicrobial drug resistance (Surveillance for Multicenter Antimicrobial Resistance in Taiwan).

### References

1. Dance DAB. Melioidosis: the tip of the iceberg? Clin Microbiol Rev 1991;4:52-60.
2. Kanaphun P, Thirawattanasuk N, Suputtamongkol Y, Naigowit P, Dance DAB, Smith MD, et al. Serology and carriage of *Pseudomonas pseudomallei*: a prospective study in 1000 hospitalized children in Northern Thailand. Clin Infect Dis 1993;167:230-3.
3. Everett ED, Nelson RA. Pulmonary melioidosis: observation in thirty-nine cases. Am Rev Respir Dis 1975;112:331-40.
4. Chaowagul W, White NJ, Dance DAB, Wattanagoon Y, Naigowit P, Davis TME, et al. Melioidosis: a major cause of community-acquired septicemia in northeastern Thailand. J Infect Dis 1989;159:890-9.
5. Smith MD, Wuthiekanun V, Walsh AL, White NJ. Quantitative recovery of *Burkholderia pseudomallei* from soil in Thailand. Trans R Soc Trop Med Hyg 1995;89:488-90.
6. Parry CM, Wuthiekanun V, Hoa NTT, Diep TS, Thao LTT, Loc PV, et al. Melioidosis in southern Vietnam: clinical surveillance and environmental sampling. Clin Infect Dis 1999;29:1323-6.
7. Yee KC, Lee MK, Chua CT, Puthucheary SD. Melioidosis, the great mimicker: a report of 10 cases from Malaysia. J Trop Med Hyg 1988;91:249-54.
8. Wilks D, Jacobson SK, Lever AML, Farrington M. Fatal melioidosis in a tourist returning from Thailand. J Infect 1994;29:87-90.
9. Smith MD, Angus BJ, Wuthiekanun V, White NJ. Arabinose assimilation defines a nonvirulent biotype of *Burkholderia pseudomallei*. Infect Immun 1997;65:4319-21.
10. Lertpatanauwun N, Sermsri K, Petkaseam A, Trakulsomboon S, Thamlikitkul V, Suputtamongkol Y. Arabinose-positive *Burkholderia pseudomallei* infection in humans. Clin Infect Dis 1999;28:927-8.
11. Silbermann MH, Gyssens IC, Endtz HP, Kuijper EJ, van der Meer JTM. Two patients with recurrent melioidosis after prolonged antibiotic therapy. Scand J Infect Dis 1997;29:199-201.
12. Mays EE, Ricketts EA. Melioidosis: recrudescence associated with bronchogenic carcinoma twenty-six years following initial geographic exposure. Chest 1975;68:261-3.
13. Lee N, Wu JL, Lee CH, Tsai WC. *Pseudomonas pseudomallei* infection from drowning: the first reported case in Taiwan. J Clin Microbiol 1985;23:352-4.
14. Lee SSJ, Liu YC, Chen YS, Wann SR, Wang JH, Yen MY, et al. Melioidosis: two indigenous cases in Taiwan. J Formos Med Assoc 1996;95:562-6.
15. Lee SSJ, Liu YC, Wang JH, Wann SR. Mycotic aneurysm due to *Burkholderia pseudomallei*. Clin Infect Dis 1998;26:1013-4.
16. Tsai WC, Liu YC, Yen MY, Wang JH, Chen YS, Wang JH, et al. Septicemic melioidosis in southern Taiwan: a case report. J Microbiol Immunol Infect 1998;31:137-40.
17. Lee SC, Ling TS, Chen JC, Huang BY, Sheih WB. Melioidosis with adrenal gland abscess. Am J Trop Med Hyg 1999;61:34-6.
18. Chen YH, Peng CF, Hwang KP, Tsai JJ, Lu PL, Chen TP. An indigenous melioidosis: a case report. Kaohsiung Journal of Medical Sciences 1999;15:292-6.
19. Dance DAB, Wuthiekanun V, Naigowit P, White NJ. Identification of *Pseudomonas pseudomallei* in clinical practice: use of simple screening tests and API 20NE. J Clin Pathol 1989;42:645-8.
20. Gilligan PH, Whittier S. *Burkholderia*, *Stenotrophomonas*, *Ralstonia*, *Brevundimonas*, *Comamonas*, and *Acidovorax*. In: Murray PR, Baron EJ, Pfaller MA, Tenover FC, Yolken RH, editors. Manual of clinical microbiology. 7th ed. Washington: American Society for Microbiology; 1999.
21. National Committee for Clinical Laboratory Standards. Performance standards for antimicrobial susceptibility testing: ninth informational supplement M100-S10. Wayne (PA): The Committee; 2000.
22. Hsueh PR, Teng LJ, Pan HJ, Chen YC, Sun CC, Ho SW, et al. Outbreak of *Pseudomonas fluorescens* bacteremia among oncology patients. J Clin Microbiol 1998;36:2914-7.

23. Hsueh PR, Teng LJ, Hung CC, Hsu JH, Yang PC, Ho SW, et al. Molecular evidence for strain dissemination of *Penicillium marneffei*: an emerging pathogen in Taiwan. J Infect Dis 2000;181:1706-12.
24. Suputtamongkol Y, Chaowagul P, Lertpatanauwun N, Intaranongpai S, Ruchutrakool T, Budhsarawong D, et al. Risk factors for melioidosis and bacteremic melioidosis. Clin Infect Dis 1999;29:408-13.
25. Mceniry DW, Gillespie SH, Felmingham D. Susceptibility of *Pseudomonas pseudomallei* to new ß-lactam and aminoglycoside antibiotics. J Antimicrob Chemother 1988;21:171-5.
26. Smith MD, Wuthiekanun V, Walsh AL, White NJ. In-vitro activity of carbapenem antibiotics against beta-lactam susceptible and resistant strains of *Burkholderia pseudomallei*. J Antimicrob Chemother 1996;37:611-5.
27. White NJ, Dance DAB, Chaowagul W, Wattanagoon Y, Wuthiekanun V, Pitakwatchara N. Halving of mortality of severe melioidosis by ceftazidime. Lancet 1989;2:697-701.
28. Chaowagul P, Simpson AJH, Suputtamongkol Y, White NJ. Empirical cephalosporin treatment of melioidosis. Clin Infect Dis 1999;28:1328.
29. Simpson AJH, Suputtamongkol Y, Smith MD, Angus BJ, Rajamuwong A, Wuthiekanun V, et al. Comparison of imipenem and ceftazidime as therapy for severe melioidosis. Clin Infect Dis 1999;29:381-7.
30. Dance DAB, Wuthiekanun V, Chaowagul W, Suputtamongkol Y, White NJ. Development of resistance to ceftazidime and co-amoxiclav in *Pseudomonas pseudomallei*. J Antimicrob Chemother 1991;29:408-13.
31. Chaowagul W, Simpson AJH, Suputtamongkol Y, Smith MD, Angus BJ, White NJ. A comparison of chloramphenicol, trimethoprim-sulfamethoxazole, and doxycycline with doxycycline alone as maintenance therapy for melioidosis. Clin Infect Dis 1999;29:375-80.
32. Chaowagul W, Suputtamongkol Y, Dance DA, Rajchanuvong A, Pattara-Arechachai J, White NJ. Relapse in melioidosis: incidence and risk factors. J Infect Dis 1993;168:1181-5.
33. Desmarchelier PM, Dance DAB, Chaowagul W, Suputtamongkol Y, White NJ, Pitt TL. Relationships among *Pseudomonas pseudomallei* isolates from patients with recurrent melioidosis. J Clin Microbiol 1993;31:1592-6.
34. Vadivelu J, Puthucheary SD, Drasar BS, Dance DAB, Pitt TL. Stability of strain genotypes of *Burkholderia pseudomallei* from patients with single and recurrent episodes of melioidosis. Trop Med Int Health 1998;3:518-21.
35. Haase A, Melder A, Smith-Vaughan H, Kemp D, Currie B. RAPD analysis of isolates of *Burkholderia pseudomallei* from patients with recurrent melioidosis. Epidemiol Infect 1995;115:115-21.
36. Trakulsomboon S, Dance DAB, Smith MD, White NJ, Pitt TL. Ribotype differences between clinical and environmental isolates of *Burkholderia pseudomallei.* J Med Microbiol 1997;46:565-70.
37. van Phung L, Chi TTB, Hotta H, Yabuuchi E, Yano I. Cellular lipid and fatty compositions of *Burkholderia pseudomallei* strains isolated from human and environment in Viet Nam. Microbiol Immunol 1995;39:105-16.
